# Clinical Use of Mental Health Digital Therapeutics in a Large Health Care Delivery System: Retrospective Patient Cohort Study and Provider Survey

**DOI:** 10.2196/56574

**Published:** 2024-10-02

**Authors:** Samuel J Ridout, Kathryn K Ridout, Teresa Y Lin, Cynthia I Campbell

**Affiliations:** 1 Department of Psychiatry, San Jose Medical Center, The Permanente Medical Group, San Jose, CA, United States; 2 Kaiser Permanente Northern California Division of Research, 4480 Hacienda Drive, Pleasanton, CA, 94588, United States, 1 650 380 2507; 3 Department of Psychiatry, Santa Rosa Medical Center, The Permanente Medical Group, Santa Rosa, CA, United States; 4 Department of Psychiatry and Behavioral Sciences, University of California San Francisco, San Francisco, CA, United States

**Keywords:** digital therapeutics, depression, anxiety, mental health, retrospective cohort, electronic health record, adults, survey, recommendation, mobile phone

## Abstract

**Background:**

While the number of digital therapeutics (DTx) has proliferated, there is little real-world research on the characteristics of providers recommending DTx, their recommendation behaviors, or the characteristics of patients receiving recommendations in the clinical setting.

**Objective:**

The aim of this study was to characterize the clinical and demographic characteristics of patients receiving DTx recommendations and describe provider characteristics and behaviors regarding DTx.

**Methods:**

This retrospective cohort study used electronic health record data from a large, integrated health care delivery system. Demographic and clinical characteristics of adult patients recommended versus not recommended DTx by a mental health provider between May 2020 and December 2021 were examined. A cross-sectional survey of mental health providers providing these recommendations was conducted in December 2022 to assess the characteristics of providers and recommendation behaviors related to DTx. Parametric and nonparametric tests were used to examine statistical significance between groups.

**Results:**

Of 335,250 patients with a mental health appointment, 53,546 (16%) received a DTx recommendation. Patients recommended to DTx were younger, were of Asian or Hispanic race or ethnicity, were female, were without medical comorbidities, and had commercial insurance compared to those without a DTx recommendation (*P*<.001). More patients receiving a DTx recommendation had anxiety or adjustment disorder diagnoses, but less had depression, bipolar, or psychotic disorder diagnoses (*P*<.001) versus matched controls not recommended to DTx. Overall, depression and anxiety symptom scores were lower in patients recommended to DTx compared to matched controls not receiving a recommendation, although female patients had a higher proportion of severe depression and anxiety scores compared to male patients. Provider survey results indicated a higher proportion of nonprescribers recommended DTx to patients compared to prescribers (*P*=.008). Of all providers, 29.4% (45/153) reported using the suggested internal electronic health record–based tools (eg, smart text) to recommend DTx, and of providers recommending DTx resources to patients, 64.1% (98/153) reported they follow up with patients to inquire on DTx benefits. Only 38.4% (58/151) of respondents report recommending specific DTx modules, and of those, 58.6% (34/58) report following up on the impact of these specific modules.

**Conclusions:**

DTx use in mental health was modest and varied by patient and provider characteristics. Providers do not appear to actively engage with these tools and integrate them into treatment plans. Providers, while expressing interest in potential benefits from DTx, may view DTx as a passive strategy to augment traditional treatment for select patients.

## Introduction

The digital therapeutics (DTx) market has experienced rapid growth in recent years, with the global DTx market valued around US $5.2 billion in 2022 and market research analysis projecting double-digit compounded annual growth from 2023 through 2030 [1]. In the first half of 2021 alone, the DTx business sector raised US $1.6 billion in venture capital [2]. Evidence suggests that DTx resources may be acceptable and scalable and therefore are a logical adjunct for mental health treatment, particularly given the growing need for mental health care [3].

However, despite DTx proliferation and potential for application in the mental health care space, patient engagement has been minimal [4]. Reasons for this poor engagement may include a lack of personal support in DTx (ie, integration of the tool into treatment rather than passive deployment of digital resources), which has been shown as important for the acceptability and usability of mental health DTx [5]. Further, there is a notable evidence gap for DTx’s mental health treatment effectiveness, with most evidence-based DTx having minimal market share [6], which may impact provider recommendations of DTx. Small, randomized controlled trials (RCTs) of DTx have shown small to moderate effect sizes for smartphone interventions in reducing depression and anxiety symptoms compared to controls [7,8]. A recent meta-analysis of 22 RCTs of mindfulness DTx showed small to moderate positive effects on depression and stress symptoms but not on anxiety [9]. An RCT of 88 college students using the Calm app (CALM.com, Inc) showed an improvement in perceived stress, mindfulness, and self-compassion in the users compared to controls [10]. Finally, an RCT of 146 employees with depressive symptoms using the myStrength app (Livongo Health, Inc) showed a more rapid reduction in depressive symptoms compared to controls [11]. The literature is not yet robust on which clinical populations are most appropriate for DTx, but those with mild to moderate symptoms of depression and anxiety, rather than severe, appear to benefit most [12,13]. DTx that are based on cognitive behavioral therapy may also be more effective, but much depends on the patient population as well as DTx design [13].

A mental health provider recommendation could be an influential factor for patient engagement with DTx tools. A 2016 survey of physicians found broad interest in DTx, but physicians also reported concerns over limited experience with such tools as well as lack of DTx data availability in the electronic medical record as barriers to DTx implementation [14]. Provider lack of knowledge regarding DTx, lack of investment and infrastructure, technical issues, workflow, and workload concerns have been identified as barriers to mobile health app adoption and implementation by providers [15-17]. In mental health, DTx tool adoption may vary by provider type and scope of practice, with psychiatrists reporting less bandwidth to focus on therapeutic DTx tools and therapists reporting that caseload, familiarity with DTx content and evidence, and limits on access to DTx data impact the ability to engage with patients around these tools [18]. Despite these descriptions of barriers to DTx tool adoption, there are little data on who providers recommend DTx tools to, the characteristics of providers recommending DTx tools, or their behaviors in recommending DTx tools. Understanding the characteristics of providers and their behaviors in recommending DTx tools can inform how these tools are integrated into mental health care, which is of particular importance given the increasing patient need for mental health resources and the dominant role mental health is assuming in the DTx space.

This study sought to characterize the clinical use of DTx resources within a large, integrated health care delivery system in two ways: (1) using electronic health record (EHR) data to identify the clinical and demographic characteristics of patients receiving recommendations from mental health providers to use DTx tools and (2) using an anonymous provider survey to determine characteristics and DTX recommending behaviors of mental health providers in clinical practice. Findings can inform the integration of DTx into mental health clinical care.

## Methods

### Setting

Kaiser Permanente Northern California (KPNC) is a large, diverse, integrated health care delivery system with approximately 4.6 million members. The membership is largely representative of the region, with less representation at the extreme ends of the income distribution [19]. Mental health services are primarily provided internally, both individually and in group format. Services are also available by referral to external community providers as needed.

### DTx Resources

KPNC offers multiple DTx tools to its members at no cost. During the study time frame, the portfolio included Calm, myStrength, Headspace (Headspace Health, Inc), SilverCloud (Amwell), Thrive (Waypoint), and Whil (RethinkFirst) [20]. Providers can recommend DTx tools to patients based on their clinical judgment using smart text elements in the EHR, which are inserted into electronic secure messages to the patients with an associated link to the DTx. This electronic secure message contact is documented in the after-visit summary if providers use these smart text elements.

### Study Design and Data Sources

#### Overview

This study had 2 components. The first used a retrospective cohort design with KPNC EHR data to identify adult patients who were seen in the mental health department, diagnosed with a mental health disorder, and received a provider recommendation to a DTx, as well as a matched cohort of patients who were also seen in the mental health department with a diagnosed mental health disorder but did not receive a recommendation to a DTx. The second component consisted of an anonymous web-based survey of health system mental health providers. This study followed the STROBE (Strengthening the Reporting of Observational Studies in Epidemiology) guidelines for observational studies [21] and the CHERRIES (Checklist for Reporting Results of Internet E-Surveys) for web-based surveys (Checklists 1 and 2) [22].

#### EHR Cohort

Adult patients aged 18 years or older with a mental health department visit and mental health diagnosis between May 1, 2020, and December 31, 2021, were included (Multimedia Appendix 1). Qualifying mental health disorders based on the *International Classification of Diseases, 10th Revision* (*ICD-10*) diagnosis codes included generalized anxiety disorder, bipolar disorder, major depressive disorder, adjustment disorders, posttraumatic stress disorder, psychosis, and sleep disorders (Multimedia Appendix 2). The index date was the first mental health visit; subsequent visits were excluded. Patients were excluded if they had less than 1-year health plan membership prior to their index mental health visit, in order to ensure the ability to capture baseline diagnoses assigned at patient encounters.

#### EHR Measures

The primary outcomes were the number and proportion of patients who received a DTx recommendation within 30 days of their index date. Within this time frame, we assumed that the primary diagnosis of their index mental health visit was the reason for a DTx recommendation. The recommendation was determined through text string searches (Multimedia Appendix 3) using relevant phrases within provider progress notes, smart data element capturing questionnaires, and secure messages from the patient’s provider.

Additional EHR-based measures included patient demographic variables (sex, race, ethnicity, and age group), type of psychiatric provider seen (nurse, therapist, and physician), neighborhood deprivation index (as a proxy measure for socioeconomic status, categorized into quintiles), insurance type (commercial, Medicare, or Medicaid), Charlson Comorbidity Index [23], and KPNC medical service area. The Patient Health Questionnaire 9-Item (PHQ-9) [24] and Generalized Anxiety Disorder 7-Item (GAD-7) [25] questionnaires, which identify symptoms for depression and anxiety, respectively, were extracted from the EHR if available within 30 days of the index visit. Each item in the PHQ-9 is scored 0 to 3, providing a 0 to 27 severity total score. The total score is used to determine cut points for depression: 0‐4 is normal, 5‐9 is mild, 10‐14 is moderate, 15‐19 is moderate severe, and 20 or more is severe.  The GAD-7 is a 7-item generalized anxiety disorder scale. Each item is scored 0 to 3, providing a 0 to 21 severity total score. The total score is used to determine cut points for generalized anxiety disorder: 0‐4 is normal, 5‐9 is mild, 10‐14 is moderate, and 15 or more is severe. 

#### Provider Survey

We conducted an anonymous web-based survey of a convenience sample of mental health providers throughout the KPNC region to understand the characteristics of providers recommending DTx tools and their recommendation behaviors. This survey was distributed 3 times in December 2022 to department managers and chiefs of psychiatry via an email that contained a Microsoft Forms link and a request to distribute it to clinical staff of approximately 2130 therapists and 375 psychiatrists. Participation was voluntary.

The survey included 26 multiple-choice questions and a free-text comment section (Multimedia Appendix 4). Specifically, we collected data on providers’ attitudes toward DTx adoption, DTx utility and effectiveness, preference for the type of DTx, awareness of different DTx modules, patient criteria for DTx recommendation, patterns of recommendation to DTx (self-assessment regarding manner of recommendation and monthly volume), barriers to recommendation, and demographics (professional role, practice experience, service area, work hours, sex [female or male self-identified hereafter referred to as “female” or “male”], age, race or ethnicity, and work hours). The primary outcome was the percentage of KPNC providers who reported recommending DTx.

### Ethical Considerations

This study received approval from the KPNC Institutional Review Board (1899252) and was determined exempt, with a waiver of consent for the EHR-based analyses and a waiver of written consent for the anonymized provider survey, which did not gather protected health information. No compensation was provided to the providers per health system policy.

### Statistical Analysis

#### EHR-Based Characteristics of Patients Receiving Versus Not Receiving DTx Recommendations

Demographic variables, clinical characteristics, and depression and anxiety symptom burden scores (PHQ-9 and GAD-7) were compared between patients who were and were not recommended to DTx with a series of bivariate analyses. Among those who received a DTx recommendation, we also examined the relationships of mental health symptoms by sex. To examine depression and anxiety diagnoses and symptom burden associated with DTx recommendations while accounting for potential confounding factors, we performed a frequency match to create a 1:1 matched cohort of patients without a recommendation to those recommended to DTx. Patients were matched based on a 5-year age group, sex, and geographical service area within KPNC. We then conducted bivariate analyses comparing the PHQ-9 and GAD-7 using the cases and matched patients.

#### Provider Survey

We performed descriptive statistics (eg, frequencies and bivariate tests) to characterize provider characteristics and DTx recommendations based on multiple-choice responses. Comparisons were done based on whether respondents were “prescribers” (eg, psychiatrists and nurse practitioners and able to prescribe medications to patients) or “nonprescribers” (eg, therapists such as psychologists and marriage and family therapists) to examine potential differences by provider role.

For both the EHR-based provider recommendation and survey analyses, the Pearson or Mantel-Haenszel chi-square test or Fisher exact test was used to calculate *P* values for categorical variables, the 2-tailed *t* test was used to calculate *P* values for continuous variables when the assumptions for a parametric test assumption were met, and the Mann-Whitney *U* test was applied to calculate ordinal data when the assumptions for a nonparametric test assumption was met. All item responses were used. All analyses were performed using SAS (version 9.4) for Windows and SAS Studio (version 3.81; SAS Institute Inc).

## Results

### Characteristics of Patients Recommended Versus Not Recommended DTx

Of the 335,250 adults eligible during the study time period, 16% (53,546/335,250) were recommended a DTx by a provider (Table 1). Patients recommended versus not recommended to a DTx were more likely to be younger (age 18‐44 years: 37,744/53,546, 70.5% vs 164,456/281,704, 58.4%; *P*<.001), Asian or Hispanic (20,360/53,546, 38% vs 88,827/281,704, 31.6%; *P*<.001), and female (38,338/53,546, 71.6% vs 193,063/281,704, 68.5%; *P*<.001). Additionally, there was a higher proportion of patients in the lowest (“0“) category of the Charlson Comorbidity Index (40,261/53,546, 75.2% vs 188,414,/281,704, 66.9%; *P*<.001) and a higher proportion of commercially insured patients (44,960/53,363, 84.3% vs 205,425/280,546, 73.2%; *P*<.001) among those who received recommendations versus those without a recommendation. Most DTx recommendations originated from therapy providers (48,916/53,546, 91.4%).

**Table 1.** Cohort characteristics^a^^,^^b^.

Characteristic	Overall cohort (N=335,250), n (%)	No DTx^c^ recommendation (n=281,704), n (%)	DTx recommendation (n=53,546), n (%)	*P* value
**Age (years)**				<.001
18‐44	202,200 (60.3)	164,456 (58.4)	37,744 (70.5)	
45‐64	92,245 (27.5)	79,668 (28.3)	12,577 (23.5)	
65‐79	34,920 (10.4)	31,987 (11.4)	2933 (5.5)	
80+	5885 (1.8)	5593 (2)	292 (0.5)	
**Race or ethnicity**				<.001
Asian	39,615 (11.8)	32,039 (11.4)	7576 (14.1)	
Black	26,799 (8)	22,503 (8)	4296 (8)	
Hispanic	69,572 (20.8)	56,788 (20.2)	12,784 (23.9)	
White	172,215 (51.4)	147,744 (52.4)	24,471 (45.7)	
Other	27,049 (8.1)	22,630 (8)	4419 (8.3)	
**Sex**				<.001
Female	231,401 (69)	193,063 (68.5)	38,338 (71.6)	
Male	103,849 (31)	88,641 (31.5)	15,208 (28.4)	
**Charlson Comorbidity Index**				<.001
0	228,675 (68.2)	188,414 (66.9)	40,261 (75.2)	
1	59,561 (17.8)	50,990 (18.1)	8571 (16)	
2‐3	29,133 (8.7)	25,874 (9.2)	3259 (6.1)	
≥4	17,881 (5.3)	16,426 (5.8)	1455 (2.7)	
**Neighborhood Deprivation Index** ^d^				<.001
Quintile 1	67,080 (20)	57,034 (20.3)	10,046 (18.8)	
Quintile 2	66,850 (20)	56,281 (20)	10,569 (19.7)	
Quintile 3	67,106 (20)	56,260 (20)	10,846 (20.3)	
Quintile 4	67,114 (20)	56,097 (19.9)	11,017 (20.6)	
Quintile 5	66,866 (20)	55,826 (19.8)	11,040 (20.6)	
**Provider type**				<.001
Nurse	6331 (1.9)	6196 (2.2)	135 (0.3)	
Physician	91,733 (27.4)	88,598 (31.5)	3135 (5.9)	
Therapist	192,971 (57.6)	144,055 (51.1)	48,916 (91.4)	
Unknown	30,357 (9.1)	29,861 (10.6)	496 (0.9)	
Other	13,858 (4.1)	12,994 (4.6)	864 (1.6)	
**Insurance type**				<.001
Commercial	250,385 (75)	205,425 (73.2)	44,960 (84.3)	
Medicaid	32,186 (9.6)	27,820 (9.9)	4366 (8.2)	
Medicare	50,629 (15.2)	46,703 (16.6)	3926 (7.4)	
Other	709 (0.2)	598 (0.2)	111 (0.2)	
**Mental health app**				N/A^e^
Calm only	16,133 (4.8)	N/A	16,133 (30.1)	
Calm or myStrength	3328 (1)	N/A	3328 (6.2)	
Other app or any combined	31,342 (9.3)	N/A	31,342 (58.5)	
myStrength only	2743 (0.8)	N/A	2743 (5.1)	
Missing	281,704 (84)	281,704 (100)	0 (0)	

a Provider referred members to DTx within 1 month of index mental health visit.

b DTx include Calm, myStrength, Headspace, SilverCloud, Thrive, or Whil.

c DTx: digital therapeutics.

d Neighborhood Deprivation Index quintile cut points for cohort.

e N/A: not applicable.

### Matched Analyses—Mental Health Diagnoses and Symptoms of Patients Recommended Versus Not Recommended DTx

Given the significant demographic differences between patients recommended a DTx versus those not recommended, we performed a matched analysis to compare mental health diagnoses and symptoms between these 2 groups. Patients recommended to DTx versus not recommended had higher proportions of anxiety (22,247/53,545, 41.5% vs 19,039/53,545, 35.6%) and adjustment disorders (11,024/53,545, 20.6% vs 7592/53,545, 14.2%) and lower proportions of depression (13,151/53,545, 24.6% vs 16,517/53,545, 30.8%), bipolar (588/53,545, 1.1% vs 2685/53,545, 5%), or psychotic diagnoses (173/53,545, 0.3% vs 1239/53,545, 2.3%; all *P*<.001; Table 2). Additionally, more patients recommended to DTx had PHQ-9 depressive symptom scores in the mild (5-9: 10,643/37,282, 28.5% vs 6926/25,749, 26.9%), moderate (10-14: 10,597/37,282, 28.4% vs 7104/25,749, 27.6%), and moderate severe (15-19: 7513/37,282, 20.2% vs 5115/25,749, 19.9%) ranges compared to patients not recommended to DTx (*P*<.001 for all). The distribution of GAD-7 anxiety scores was similar between patients recommended and not recommended to DTx, albeit with the distribution of recommended patients having a slightly higher proportion of mild (5-9: 4852/23,251, 20.9% vs 2579/13,080, 19.7%) and lower proportion of severe (≥15: 9283/23,251, 39.9% vs 5391/13,080, 41.2%) GAD-7 scores (*P*=.003 for all).

**Table 2.** Mental health diagnoses and symptoms of patients recommended to digital therapeutics (DTx) and a matched cohort of patients not recommended to DTx^a^^,b^.

Characteristic	Matched cohort (n=53,545), n (%)	Cases (n=53,545), n (%)	*P* value
**Age (years)**			>.99
18‐44	37,743 (70.5)	37,743 (70.5)	
45‐64	12,577 (23.5)	12,577 (23.5)	
65‐79	2933 (5.5)	2933 (5.5)	
80+	292 (0.5)	292 (0.5)	
**Race or ethnicity**			.16
Asian	7577 (14.2)	7576 (14.1)	
Black	4465 (8.3)	4296 (8)	
Hispanic	12,512 (23.4)	12,783 (23.9)	
White	24,511 (45.8)	24,471 (45.7)	
Other	4480 (8.4)	4419 (8.3)	
**Sex**			>.99
Female	38,337 (71.6)	38,337 (71.6)	
Male	15,208 (28.4)	15,208 (28.4)	
**Charlson Comorbidity Index**			<.001
0	39,053 (72.9)	40,260 (75.2)	
1	9114 (17)	8571 (16)	
2‐3	3575 (6.7)	3259 (6.1)	
≥4	1803 (3.4)	1455 (2.7)	
**Neighborhood Deprivation Index** ^c^			<.001
Quintile 1	9337 (17.4)	10,045 (18.8)	
Quintile 2	9840 (18.4)	10,569 (19.7)	
Quintile 3	10,764 (20.1)	10,846 (20.3)	
Quintile 4	11,355 (21.2)	11,017 (20.6)	
Quintile 5	12,219 (22.8)	11,040 (20.6)	
**Provider type**			<.001
Nurse	1011 (1.9)	135 (0.3)	
Physician	13,224 (24.7)	3135 (5.9)	
Therapist	36,812 (68.7)	48,916 (91.4)	
Unknown	625 (1.2)	495 (0.9)	
Other	1873 (3.5)	864 (1.6)	
**Insurance type^d^**			<.001
Commercial	42,830 (80.3)	44,959 (84.3)	
Medicaid	5498 (10.3)	4366 (8.2)	
Medicare	4870 (9.1)	3926 (7.4)	
Other	124 (0.2)	111 (0.2)	
**Mental health disorder diagnosis at visit**			<.001
Anxiety	19,039 (35.6)	22,247 (41.5)	
Bipolar	2685 (5)	588 (1.1)	
Depression	16,517 (30.8)	13,151 (24.6)	
Other mood	7592 (14.2)	11,024 (20.6)	
Posttraumatic stress disorder	6441 (12)	6341 (11.8)	
Psychosis	1239 (2.3)	173 (0.3)	
Sleep	32 (0.1)	21 (0)	
**Mental health symptom PHQ-9** ^**d**^ ^**,** **e**^			<.001
Normal	3541 (13.8)	4063 (10.9)	
Mild	6926 (26.9)	10,643 (28.5)	
Moderate	7104 (27.6)	10,597 (28.4)	
Moderate severe	5115 (19.9)	7513 (20.2)	
Severe	3063 (11.9)	4466 (12)	
**Mental health symptom GAD-7** ^**d**^ ^**,** **f**^			.003
Normal	508 (3.9)	857 (3.7)	
Mild	2579 (19.7)	4852 (20.9)	
Moderate	4602 (35.2)	8259 (35.5)	
Severe	5391 (41.2)	9283 (39.9)	

a Provider referred members to DTx within 1 month of index mental health visit.

b DTx includes Calm, myStrength, Headspace, SilverCloud, Thrive, or Whil.

c Neighborhood Deprivation Index reports quintile cut points for our specific study population.

d Section does sum to a cohort of recommended patients because of missing item data.

e Patient Health Questionnaire 9-Item depression scale. Each item is scored 0 to 3, providing a 0 to 27 severity total score. Using the total score to represent the cut point for depression, 0‐4 is normal, 5‐9 is mild, 10‐14 is moderate, 15‐19 is moderate severe, and 20 or more is severe.

f Generalized Anxiety Disorder 7-Item scale. Each item is scored 0 to 3, providing a 0 to 21 severity total score. Using the total score to represent the cut point for generalized anxiety disorder, 0‐4 is normal, 5‐9 is mild, 10‐14 is moderate, and 15 or more is severe.

### Mental Health Symptom Burden by Sex in DTx-Recommended Patients

Since approximately two-thirds of patients recommended to DTx were female, we conducted ad hoc analyses examining symptom burden stratified by sex among those who received a recommendation to DTx. Female patients recommended to DTx had higher proportions of PHQ-9 depressive symptom scores in the moderate (7575/26,416, 28.7% vs 3022/10,867, 27.8%), moderate severe (5414/26,416, 20.5% vs 2099/10,867, 19.3%), and severe (3246/26,416, 12.3% vs 1220/10,867, 11.2%) range compared to male patients; and male patients had higher proportions of PHQ-9 scores in the normal (2804/26,416, 10.6% vs 1260/10,867, 11.6%) and mild (7377/26,416, 27.9% vs 3266/10,867, 30.1%) ranges (*P*=.003; Table 3). Female patients recommended to DTx had higher proportions of GAD-7 scores in the severe (6839/16,526, 41.4% vs 2444/6725, 36.3%) range compared to male patients; and male patients had more representation in the normal (516/16,526, 3.1% vs 341/6725, 5.1%) and mild (3296/16,526, 19.9% vs 1556/6725, 23.1%) ranges for GAD-7 (*P*<.001).

**Table 3.** Mental health symptoms by sex among patients recommended digital therapeutics (DTx)^a,b^.

Characteristic	Overall cohort (N=53,546), n (%)	Female patients (n=38,337), n (%)	Male patients (n=15,208), n (%)	*P* value
**Mental health symptom PHQ-9** ^c d^				.003
Normal	4064 (10.90)	2804 (10.6)	1260 (11.6)	
Mild	10,643 (28.55)	7377 (27.9)	3266 (30.1)	
Moderate	10,597 (28.42)	7575 (28.7)	3022 (27.8)	
Moderate severe	7513 (20.15)	5414 (20.5)	2099 (19.3)	
Severe	4466 (11.98)	3246 (12.3)	1220 (11.2)	
**Mental health symptom GAD-7** ^d e^				<.001
Normal	857 (3.69)	516 (3.1)	341 (5.1)	
Mild	4852 (20.87)	3296 (19.9)	1556 (23.1)	
Moderate	8259 (35.52)	5875 (35.6)	2384 (35.4)	
Severe	9283 (39.93)	6839 (41.4)	2444 (36.3)	

a Member recommended DTx within 1 month of index mental health visit.

b DTx includes Calm, myStrength, Headspace, SilverCloud, Thrive, or Whil.

c Patient Health Questionnaire 9-Item depression scale. Each item is scored 0 to 3, providing a 0 to 27 severity total score. Using the total score to represent the cut point for depression, 0‐4 is normal, 5‐9 is mild, 10‐14 is moderate, 15‐19 is moderate severe, and 20 or more is severe.

d Columns do not sum to the respective cohort because of missing data.

e Generalized Anxiety Disorder 7-Item scale. Each item is scored 0 to 3, providing a 0 to 21 severity total score. Using the total score to represent the cut point for generalized anxiety disorder, 0‐4 is normal, 5‐9 is mild, 10‐14 is moderate, and 15 or more is severe.

### Characteristics of Providers Recommending DTx

A total of 211 individuals responded to the provider survey on DTx prescribing patterns for a participation rate of 8.4% (211/2505). Of those, 19.6% (40/204) identified as prescribers, 68.6% (140/204) as nonprescribers, and the remainder as having an administrative or managerial role in the clinic. Of respondents, 91.9% (192/209) were between the ages of 30 and 59 years, 54.1% (113/209) identified as White, 17.2% (36/209) as Asian, 12% (25/209) as Hispanic or Latino, and 6.2% (13/209) as Black. Nearly all respondents reported working at least half-time, and two-thirds of respondents reported working a full-time schedule.

### Provider DTx Recommendation Behaviors

Overall, a higher proportion of nonprescribers (127/164, 77.4%) indicated they recommend DTx to patients compared to prescribers (22/39, 56.4%; *P*=.008). Of providers who recommend DTx, 83.6% (122/146) do so via an electronic secure message to patients, and only 12.3% (18/146) do so during a clinic appointment. Calm was the most commonly recommended DTx, followed by myStrength, Headspace, SilverCloud, Thrive, and Whil. Only 29.4% (45/153) of providers indicated using the suggested internal EHR–based smart text to recommend DTx. Prescribers were more likely than nonprescribers to report that they did not know how to make patient recommendations using EHR-based protocols (*P*=.01). Of providers recommending DTx to patients, 64.1% (98/153) report that they follow up with patients to inquire whether the resource was helpful. Only 38.4% (58/151) of providers recommend specific modules of DTx resources, and of those 58.6% (34/58) report following up with patients on these specific recommendations. On a scale of 0‐10 regarding the perceived effectiveness of DTx resources to impact symptoms (0=none and 10=extreme), both prescribers and nonprescribers rated cognitive behavioral therapy and mindfulness content between 6 and 7 or moderate to highly effective.

## Discussion

### Principal Findings

These are some of the first pragmatic results regarding provider recommendation patterns, characteristics, and attitudes toward mental health DTx. In this large, integrated health system with mental health DTx tools included as a benefit, a very modest percentage of patients with mental health disorders received recommendations to DTx tools. Those who did were more likely to be young, female, Asian or Hispanic, with less medical comorbidity, and to have commercial insurance compared to patients not recommended to DTx. Recommendations for DTx were more often made for anxiety or adjustment diagnoses and for patients with less severe mental health symptoms, although female patients recommended to DTx tools had a higher proportion of anxiety symptoms compared to male patients. A provider survey showed that DTx are recommended more frequently by nonprescribers and via electronic secure messages rather than during clinical appointments. Of providers recommending DTx, a modest majority follow up with the patient regarding DTx effectiveness, and few knew how to follow the recommended EHR protocols to do so.

Previous studies reporting demographic characteristics of patients using DTx have been from pilot trials or RCTs [7-9]. Using real-world data collected in the course of clinical care, we found that patient characteristics were similar to those of DTx trials. Overall, patients who received DTx recommendations were younger with less severe mental health symptoms, suggesting that providers are likely tailoring their recommendations to those they feel are most suitable. This may also reflect the initial emphasis of the health system DTx implementation focusing on patients with mild to moderate symptoms of depression or anxiety [20]. These results highlight a gap in the evidence base regarding the effectiveness of DTx for patients with mild to severe mental health symptoms or multiple comorbidities.

Asian or Hispanic patients were more likely to receive DTx recommendations. There are many potential drivers of this finding, including provider beliefs about patient DTx uptake, patient ability to interface with DTx, if providers felt the symptom severity reported by these patients was more appropriate for DTx, or other unrecognized factors. This finding merits further investigation. Of note, the significant association for insurance type with a DTx recommendation suggests slightly more patients with commercial insurance receiving recommendations. This may reflect provider-perceived barriers to care (eg, differences in disease severity or mobile phone access) for patients with other types of insurance coverage.

The finding that a higher proportion of female patients with severe mental health symptoms were referred to DTx compared to male patients could reflect a bias to implement more tools to treat symptoms in female patients, a reporting bias of symptoms by male or female patients, refusal of the DTx recommendation by male patients, or other sex-associated biases in care [26]. These findings warrant further research.

Provider survey results indicate that a higher proportion of nonprescribers recommend DTx and reported familiarity with EHR-based protocols to recommend DTx compared to prescribers. These results could reflect uneven training by provider type during the initial rollout or a lack of awareness or interest in the DTx tools by prescribers. Nonprescribers may appreciate having another treatment option to recommend beyond their typical strategies, given the therapeutic role DTx can fill and the silos of mental health care (ie, “therapy” vs “medication management” tasks). Interestingly, a minority of providers reported using the EHR-based protocols to recommend DTx to patients, with very few recommending specific modules or follow-up on module recommendations. Providers may view DTx tools as a passive, adjunctive resource rather than part of an active mental health treatment plan. Further, only a modest majority of providers reported asking patients if they found the DTx effective. A similar follow-up rate for a recommended or prescribed medical device, medication, or therapeutic program such as intensive outpatient or partial hospitalization would likely be regarded as very low. It is unclear why providers perceive DTx offerings in this way; it is possible that providers do not see DTx as having robust effectiveness data, which may impact their rate of follow-up. Similarly, providers may not view DTx resources as part of clinical care or within their scope of practice to monitor. This may reflect the early stage of growth and implementation of DTx in mental health and may evolve over time. If DTx are to be integrated into clinical mental health care, it may be more effective as a formalized part of the treatment plan and followed in the same way as other treatment aspects.

### Limitations

This study was conducted in an integrated health care system, which limits generalizability to providers and patients in different systems of care. The survey participation rate was low, given constraints related to direct dissemination and reapproaching for response, though consistent with reported response rates in similar investigations [15]. While our integrated health system made multiple DTx available at no cost to patients during the study time period, such resources may not be as easily available to patients in other health care systems. Recommendations that were in person only or sent in messages without using the EHR protocols and smart text were not captured, nor were recommendations related to later visits, although our intent was to identify options presented earlier in the treatment course. The large sample size of EHR-based recommendation data makes it likely to detect associations that may have statistical significance but may not have meaningful clinical implications. The data we explored are observational and exploratory; future work warrants further examination into these initial findings along with an examination of patient-level outcomes associated with DTx.

### Conclusions

The use of DTx is growing at a rapid pace, and health systems and patients see these as potentially valuable resources. However, the extent of provider recommendations to DTx is modest, as is provider knowledge about DTx and their follow-up with patients about DTx use, which suggests a largely passive DTx uptake by providers. Given the likely continued interest by patients, health care systems, and industry, future research on how to effectively implement these tools is critical.

## Supplementary material

10.2196/56574 Multimedia Appendix 1 Cohort of Kaiser Permanente Northern California psychiatry patients receiving digital therapeutics recommendation.

10.2196/56574 Multimedia Appendix 2 *International Classification of Diseases* codes for mental health conditions.

10.2196/56574 Multimedia Appendix 3 Data sources and search terms used to capture digital health recommendations.

10.2196/56574 Multimedia Appendix 4 Provider survey.

10.2196/56574 Checklist 1 STROBE (Strengthening the Reporting of Observational Studies in Epidemiology) checklist.

10.2196/56574 Checklist 2 CHERRIES (Checklist for Reporting Results of Internet E-Surveys).

## Abbreviations

CHERRIES: Checklist for Reporting Results of Internet E-Surveys
DTx: digital therapeutics
EHR: electronic health record
GAD-7: Generalized Anxiety Disorder 7-Item
*ICD-10*: *International Classification of Diseases, 10th Revision*
KPNC: Kaiser Permanente Northern California
PHQ-9: Patient Health Questionnaire 9-Item
RCT: randomized controlled trial
STROBE: Strengthening the Reporting of Observational Studies in Epidemiology

## References

[1] (2023). Digital transformation market size, share & trends analysis report by solution (analytics, mobility), by deployment, by service, by enterprise size, by end-use, by region, and segment forecasts, 2023 - 2030. Grand View Research.

[2] (2023). 1H and Q2 2021 digital health funding and M&A report. Mercom Capital Group.

[3] Zhong S, Yang X, Pan Z (2023). The usability, feasibility, acceptability, and efficacy of digital mental health services in the COVID-19 pandemic: scoping review, systematic review, and meta-analysis. JMIR Public Health Surveill.

[4] Aziz M, Erbad A, Almourad MB, Altuwairiqi M, McAlaney J, Ali R (2022). Did usage of mental health apps change during COVID-19? A comparative study based on an objective recording of usage data and demographics. Life (Basel).

[5] Patel S, Akhtar A, Malins S (2020). The acceptability and usability of digital health interventions for adults with depression, anxiety, and somatoform disorders: qualitative systematic review and meta-synthesis. J Med Internet Res.

[6] Satre DD, Meacham MC, Asarnow LD, Fisher WS, Fortuna LR, Iturralde E (2021). Opportunities to integrate mobile app-based interventions into mental health and substance use disorder treatment services in the wake of COVID-19. Am J Health Promot.

[7] Firth J, Torous J, Nicholas J (2017). The efficacy of smartphone-based mental health interventions for depressive symptoms: a meta-analysis of randomized controlled trials. World Psychiatry.

[8] Firth J, Torous J, Nicholas J, Carney R, Rosenbaum S, Sarris J (2017). Can smartphone mental health interventions reduce symptoms of anxiety? A meta-analysis of randomized controlled trials. J Affect Disord.

[9] Tan ZYA, Wong SH, Cheng LJ, Lau ST (2022). Effectiveness of mobile-based mindfulness interventions in improving mindfulness skills and psychological outcomes for adults: a systematic review and meta-regression. Mindfulness.

[10] Huberty J, Green J, Glissmann C, Larkey L, Puzia M, Lee C (2019). Efficacy of the mindfulness meditation mobile app “Calm” to reduce stress among college students: randomized controlled trial. JMIR Mhealth Uhealth.

[11] Hirsch A, Luellen J, Holder JM (2017). Managing depressive symptoms in the workplace using a web-based self-care tool: a pilot randomized controlled trial. JMIR Res Protoc.

[12] Koh J, Tng GYQ, Hartanto A (2022). Potential and pitfalls of mobile mental health apps in traditional treatment: an umbrella review. J Pers Med.

[13] Linardon J, Torous J, Firth J, Cuijpers P, Messer M, Fuller-Tyszkiewicz M (2024). Current evidence on the efficacy of mental health smartphone apps for symptoms of depression and anxiety. a meta-analysis of 176 randomized controlled trials. World Psychiatry.

[14] Kong T, Scott MM, Li Y, Wichelman C (2020). Physician attitudes towards—and adoption of—mobile health. Digit Health.

[15] Sezgin E, Özkan-Yildirim S, Yildirim S (2017). Investigation of physicians’ awareness and use of mHealth apps: a mixed method study. Health Policy Technol.

[16] van Kessel R, Roman-Urrestarazu A, Anderson M (2023). Mapping factors that affect the uptake of digital therapeutics within health systems: scoping review. J Med Internet Res.

[17] Borges do Nascimento IJ, Abdulazeem H, Vasanthan LT (2023). Barriers and facilitators to utilizing digital health technologies by healthcare professionals. NPJ Digit Med.

[18] Dahlhausen F, Zinner M, Bieske L, Ehlers JP, Boehme P, Fehring L (2021). Physicians’ attitudes toward prescribable mHealth apps and implications for adoption in Germany: mixed methods study. JMIR Mhealth Uhealth.

[19] Gordon N (2020). Similarity of adult Kaiser Permanente members to the adult population in Kaiser Permanente’s Northern California service area: comparisons based on the 2017/2018 cycle of the California Health Interview Survey. Kaiser Permanente Division of Research.

[20] Mordecai D, Histon T, Neuwirth E (2021). How Kaiser Permanente created a mental health and wellness digital ecosystem. NEJM Catal.

[21] von Elm E, Altman DG, Egger M (2007). The Strengthening the Reporting of Observational Studies in Epidemiology (STROBE) statement: guidelines for reporting observational studies. Epidemiology.

[22] Eysenbach G (2004). Improving the quality of web surveys: the Checklist for Reporting Results of Internet E-Surveys (CHERRIES). J Med Internet Res.

[23] Charlson ME, Pompei P, Ales KL, MacKenzie CR (1987). A new method of classifying prognostic comorbidity in longitudinal studies: development and validation. J Chronic Dis.

[24] Kroenke K, Spitzer RL, Williams JB (2001). The PHQ-9: validity of a brief depression severity measure. J Gen Intern Med.

[25] Löwe B, Decker O, Müller S (2008). Validation and standardization of the Generalized Anxiety Disorder Screener (GAD-7) in the general population. Med Care.

[26] Pattyn E, Verhaeghe M, Bracke P (2015). The gender gap in mental health service use. Soc Psychiatry Psychiatr Epidemiol.

