# Supplementary material for: Clinical Use of Mental Health Digital Therapeutics in a Large Health Care Delivery System: Retrospective Patient Cohort Study and Provider Survey
Source: JMIR Ment Health. 2024 Oct 2;11:e56574. doi: 10.2196/56574 (PMC11463191; doi:10.2196/56574)
Supplement: Multimedia Appendix 1 [file mental-v11-e56574-s001.pptx]

## Slide 1
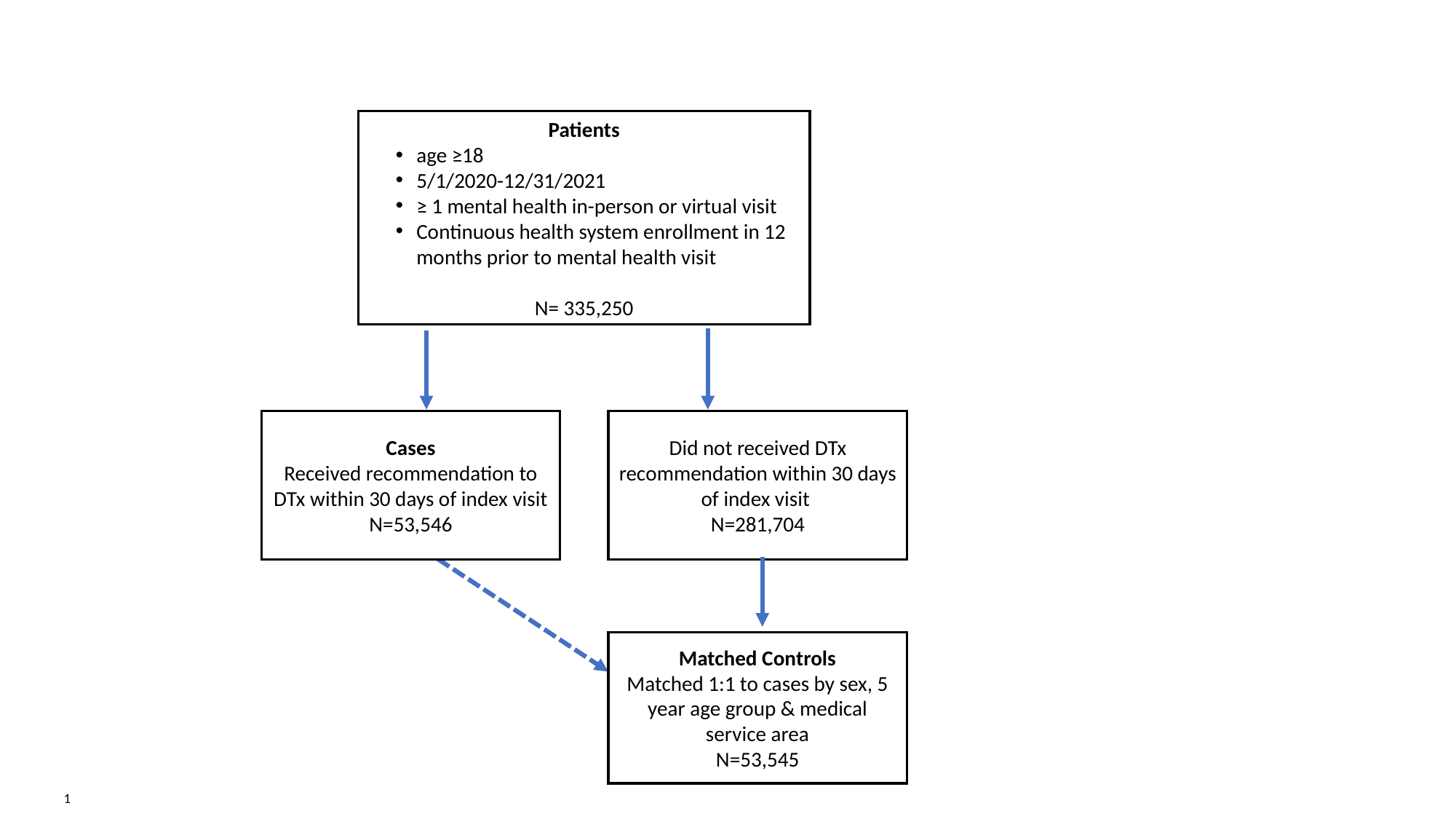

Patients
age ≥18
5/1/2020-12/31/2021
≥ 1 mental health in-person or virtual visit
Continuous health system enrollment in 12 months prior to mental health visit
N= 335,250
Cases
Received recommendation to DTx within 30 days of index visit N=53,546
Did not received DTx recommendation within 30 days of index visit
N=281,704
Matched Controls
Matched 1:1 to cases by sex, 5 year age group & medical service area
N=53,545
1
