# Supplementary material for: Clinical Use of Mental Health Digital Therapeutics in a Large Health Care Delivery System: Retrospective Patient Cohort Study and Provider Survey
Source: JMIR Ment Health. 2024 Oct 2;11:e56574. doi: 10.2196/56574 (PMC11463191; doi:10.2196/56574)
Supplement: Multimedia Appendix 2 [file mental-v11-e56574-s002.docx]

| Mental Health Condition | Diagnosis codes from ICD-10^a^ |
| --- | --- |
| Bipolar disorder | F30.xx, F31.xx, F34.0 |
| Generalized anxiety disorder | F40.xx, F41.xx,F42.xx, F43.22 |
| MDD | F32.xx, F33.xx, F34.1, F34.81, F34.89, F34.9 |
| Other mood disorders | F06.xx, F43.21, F43.23, R45.86 |
| Psychosis | F20.xx, F22.xx, F23.xx, F25.xx, F28.xx, F29.xx |
| PTSD | F43.0, F43.10, F43.11, F43.12, F43.20, F43.24, F43.25, F43.29, F43.8, F43.9 |
| Sleep disorders | F47.xx, F51.xx |

^a^ ICD-10 = International Classification of Disease 10^th^ Revision, GAD = generalized anxiety disorder, MDD = Major depressive disorder, PTSD = post-traumatic stress disorder
