# Supplementary material for: Clinical Use of Mental Health Digital Therapeutics in a Large Health Care Delivery System: Retrospective Patient Cohort Study and Provider Survey
Source: JMIR Ment Health. 2024 Oct 2;11:e56574. doi: 10.2196/56574 (PMC11463191; doi:10.2196/56574)
Supplement: Multimedia Appendix 4 [file mental-v11-e56574-s004.pdf]

Utility of Mental Health Applications (Provider Survey)

This survey involves a research study that is designed to obtain data around how providers (you and your colleagues) utilize the mobile mental health applications ('apps') available to Kaiser Permanente members.

The survey is anonymous though we do ask for information on your job role, location and other similar items in the interest of obtaining the most valuable and useful data possible. We will not cross-reference collected data with employee records at any site at anytime.

Your participation is voluntary. You can choose to skip any question you would like, and you can withdraw your participation at any time. Already collected data cannot be withdrawn. Your responses are confidential and CANNOT be directly linked to you.

There are no foreseeable risks. Any time data are collected there is a small chance that a breach of confidentiality may occur. However, we have many safeguards in place to prevent this risk. We collect data anonymously. We use software that provides a high level of protection and we store your responses behind the Kaiser Permanente firewall.

We do not expect you to benefit directly from participation. However, your responses will help us to improve mental health application utilization and clinical care.

Please answer as honestly as possible about your use (or lack thereof) of mental health apps and practice habits. There are no right or wrong answers. Thank you for your participation. By continuing with the survey, you are consenting to participate in this study. If you have any questions, please contact principal investigator, Samuel Ridout, MD/PhD at 707-571-3778. Questions about your rights as a study participant, comments or complaints about the study may be presented to the Kaiser Permanente Northern California Institutional Review Board 1800 Harrison Street, Oakland. CA 94612, or 1-866-241-0690.

1. What is your highest degree/qualification?

- ◯ MD
- ◯ DO
- ◯ PsyD
- ◯ PhD
- ◯ LCSW
- ◯ MFT
- ◯ MD/PhD
- ◯ PSYT
- ◯ MA
- ◯ RN
- ◯ LPN
- ◯ PharmD
- ◯ Other

2. How would you describe your primary clinical role?

- ◯ Therapist
- ◯ Prescriber/Medication Management
- ◯ Other

3. What location do you work in?

- ◯ Greater East Bay (ALM, ALH, BEK, OAK, PIN, RCH)
- ◯ Diablo (ANT, BSR, DUB, SHA, WCR, DRV, LVM, MTZ, MDH, PLS)
- ◯ Fresno (CLO, FRS, OKH, SEL)
- ◯ South Bay (CMB, SKP, SVM, WTV, GIL, MIL, MTN, SCB, SCH, SCL, STR)
- ◯ North Valley (FOL, LNC, RCO, RRV, ROS, SAC, SRY)
- ◯ South Sacramento (PMB, DAV, ELG, SSC)
- ◯ Napa/Solano (FLD, KFR, NAP, VAC, VAL)
- ◯ Greater Southern Alameda (FRE, HAY, UNC, SLH, SLN)
- ◯ Central Valley (MAN, MOD, STK, TRA)
- ◯ Golden Gate (DLC, NOV, PET, RPK, RWC, SFO, SMM, SRD, SRF, SRO, SSF, SFM, SMW, SRG)
- ◯ Other

4. What department do you work in?

- ◯ Psychiatry including AMRS
- ◯ AFM
- ◯ Pediatrics
- ◯ OB/GYN
- ◯ HBS
- ◯ Health Education
- ◯ Behavioral Medicine

5. What is your age?

- ◯ Under 30
- ◯ 30-39
- ◯ 40-49
- ◯ 50-59
- ◯ 60-69
- ◯ 70-79
- ◯ 80-89
- ◯ 90+

6. What is your race?

- ◯ American Indian or Alaska Native
- ◯ Asian
- ◯ Black or African American
- ◯ Hispanic/Latino
- ◯ Native Hawaiian or Other Pacific Islander
- ◯ White
- ◯ Multiracial
- ◯ Unknown
- ◯ Other

7. Please indicate the FTE you generally work per week (Physicians- just move the decimal place on the units you work/week one place to the left).

- ◯ 0.1
- ◯ 0.2
- ◯ 0.3
- ◯ 0.4
- ◯ 0.5
- ◯ 0.6
- ◯ 0.7
- ◯ 0.8
- ◯ 0.9
- ◯ 1.0

8. Please indicate your years of practice with TPMG.

- ◯ 1-5
- ◯ 6-10
- ◯ 11-15
- ◯ 16-20
- ◯ 20+

9. Do you refer patients to the mental health mobile apps?

- ◯ Yes
- ◯ No

10. What apps do you refer patients to? (Check all that apply)

- [ ] Calm
- [ ] Headspace
- [ ] myStrength
- [ ] SilverCloud
- [ ] Thrive
- [ ] Whil
- [ ] All of the above
- [ ] Other

11. Please rank the following apps in order of your preference for referring or recommending to patients when primarily addressing depression with one being the app you most prefer and five being the one you least prefer.

Calm

Headspace

myStrength

SilverCloud

Thrive

Whil

Other

12. Please rank the following apps in order of your preference for referring or recommending to patients when primarily addressing anxiety with the first one in the list being the app you most prefer and the last one being the one you least prefer.

Calm

Headspace

myStrength

SilverCloud

Thrive

Whil

Other

13. Please rank the following apps in order of your preference for referring or recommending to patients when primarily addressing PTSD and trauma symptoms with the first one in the list being the app you most prefer and the last one being the one you least prefer.

Calm

Headspace

myStrength

SilverCloud

Thrive

Whil

Other

14. Please rank the following apps in order of your preference for referring or recommending to patients when primarily addressing psychotic disorders with the first one in the list being the app you most prefer and the last one being the one you least prefer.

Calm

Headspace

myStrength

SilverCloud

Thrive

Whil

Other

15. What method do you use to refer patients to these apps?

- ◯ Secure message with own smartphrase
- ◯ Secure message using Chamai AVS text
- ◯ [ ] Other

16. Do you insert the Chamai progress note (i.e., .chamaiprogressnote) smart text into your note when making this referral?

- ◯ Yes
- ◯ No

17. Do you follow up with patients about their use and impact of apps on symptoms after making this referral?

- ◯ Yes
- ◯ No

18. Do you recommend units or modules in the app for the patient to use based on their diagnosis or other treatment planning regarding these apps?

- ◯ Yes
- ◯ No

19. Do you follow up with patients on these specific modules?

- ◯ Yes
- ◯ No

20. How do you follow up with patients?

- ☐ Secure message
- ☐ Tridiuum
- ☐ PHQ-9
- ☐ Phone appointment
- ☐ During next scheduled appointment
- ☐ __________

Other

21. Do you differentiate the apps in your referrals on whether the app is CBT-based or mindfulness-based?

- ◯ Yes
- ◯ No

22. Estimate the % of new patients (patients you have not previously seen) you refer to apps in an average month.

- ◯ 0-25
- ◯ 26-50
- ◯ 51-75
- ◯ 76-100

23. Estimate the % of return patients (patients you have previously seen) you refer to apps in an average month.

- ◯ 0-25
- ◯ 26-50
- ◯ 51-75
- ◯ 76-100

24. On a scale of 1 - 10 with 1 being "none" and 10 being "extreme", please estimate how much impact you believe CBT app use can have on symptoms (ex. depression, anxiety, trauma).

1	2	3	4	5	6	7	8	9	10
◯	◯	◯	◯	◯	◯	◯	◯	◯	◯

25. On a scale of 1 - 10 with 1 being "none" and 10 being "extreme", please estimate how much impact you believe mindfulness-based app use can have on symptoms (ex. depression, anxiety, trauma).

1	2	3	4	5	6	7	8	9	10
○	○	○	○	○	○	○	○	○	○

26. What are the barriers to recommending apps to your patients (select all that apply)?

- ☐ Time in appointments
- ☐ Difficulty integrating into treatment such as following up on modules used and impact of them
- ☐ Knowledge of how to refer patients to apps
- ☐ Knowledge of app content
- ☐ Lack of patient motivation in utilizing apps
- ☐ Clinical conditions barring app use (psychosis, substance abuse)

27. Thank you for your participation. Your contribution to helping us better understand how providers at Kaiser Permanente utilize mobile mental health applications ('apps') in their practice is invaluable. If you have any questions about accessing applications, please contact your manager. If you have any questions or concerns about participating in the survey, please contact the principal investigator, Samuel Ridout, MD/PhD at [redacted]. If you would like to elaborate on any of your answers, please do so in the box below. Questions about your rights as a study participant, comments or complaints about the study may be presented to the Kaiser Permanente Northern California Institutional Review Board 1800 Harrison Street, Oakland. CA 94612, or 1-866-241-0690.

This content is neither created nor endorsed by Microsoft. The data you submit will be sent to the form owner.

Microsoft Forms
